# Perspective: The Challenge of Clinical Decision-Making for Drug Treatment in Older People. The Role of Multidimensional Assessment and Prognosis

**DOI:** 10.3389/fmed.2014.00061

**Published:** 2015-01-14

**Authors:** Alberto Pilotto, Daniele Sancarlo, Julia Daragjati, Francesco Panza

**Affiliations:** ^1^Geriatrics Unit, Azienda ULSS 16 Padova, Hospital S. Antonio, Padova, Italy; ^2^Geriatric Unit, Laboratory of Gerontology and Geriatrics, Department of Medical Sciences, IRCCS “Casa Sollievo della Sofferenza”, San Giovanni Rotondo, Foggia, Italy; ^3^Neurodegenerative Disease Unit, Department of Basic Medicine, Neuroscience, and Sense Organs, University of Bari Aldo Moro, Bari, Italy

**Keywords:** older age, frailty, clinical decision-making, multidimensional prognostic index, comprehensive geriatric assessment

## Abstract

A complex decision path with a careful evaluation of the risk–benefit ratio is mandatory for drug treatment in advanced age. Enrollment biases in randomized clinical trials (RCTs) cause an under-representation of older individuals. In high-risk frail older subjects, the lack of RCTs makes clinical decision-making particularly difficult. Frail individuals are markedly susceptible to adverse drug reactions, and frailty may result in reduced treatment efficacy. Life expectancy should be included in clinical decision-making paths to better assess the benefits and risks of different drug treatments in advanced age. We performed a scoping review of principal hospital- and community-based prognostic indices in older age. Mortality prognostic tools could help clinical decision-making in diagnostics and therapeutics, tailoring appropriate intervention for older patients. The effectiveness of drug treatments may be significantly different in older patients with different risk of mortality. Clinicians need to consider the prognostic information obtained through well-validated, accurate, and calibrated predictive tools to identify those patients who may benefit from drug treatments given with the aim of increasing survival.

## Evidence-Based Medicine in Older Age

Drug treatment in advanced age is achieved through a complex decision path requiring a careful evaluation of the risk–benefit ratio. Age-related changes in response to drugs can arise from pharmacokinetic and pharmacodynamic differences. However, age itself is not the only factor that could affect pharmacokinetic and pharmacodynamic responses to drugs in older people. Although guidelines pertaining to the inclusion of older participants in randomized clinical trials (RCTs) have existed for more than two decades (1), different studies and systematic reviews suggested ongoing enrollment biases with under-representation of older individuals especially in clinical trials of cardiovascular diseases (2–4) and cancer (5, 6). In fact, older adults remain underrepresented in clinical trials of patients hospitalized with an acute coronary syndrome (2) or in ongoing RCTs regarding heart failure (3), while a very recent systematic review found that no RCTs of statin or any other hypocholesterolemic medication included persons older than 82 years at baseline (4). On the same vein, from 2007 to June 2010, 24 drugs were approved for the treatment of cancer, and, on average, only 33% of patients included in the registration trials were aged 65 years or older (6), down from the 36% reported from 1992 to 2002 (5).

Conducting RCTs with older adults can be challenging for various reasons including an increased prevalence of multiple comorbidities and polypharmacy. It is well known that due to these factors, the line between benefit and harm caused by drug treatments is really thin in older subjects and not easily evaluable in routine clinical practice. Indeed, numerous clinical trials excluded patients with comorbidities such as kidney failure, hypertension, and diabetes, all of which increase in prevalence with advancing age. Moreover, even in the absence of specific age- or disease-based exclusion criteria, older subjects suffering from multiple comorbidities, with polypharmacy, physical disability, functional and cognitive impairments, malnutrition, a low familiar and social care network, or a reduced life expectancy are usually excluded from RCTs (7). While the exclusion of older subjects from RCTs is often understandable from the standpoint of completing a study safely and efficiently, the results of studies that include a very narrow, highly selected subset of subjects and exclude most of the older high-risk patients may be less generalizable to patients cared outside of the context of clinical trials. This situation may lead to uncertainty and confusion in clinical decision-making of the older people.

Beyond clinical decision-making for drug treatment in older age, prognostic information can be very useful to make a decision before high-risk interventions such as major surgery (8), particularly to prevent institutionalization, a second fracture in institutionalized patients, and decrease mortality after a hip fracture (9). In fact, the decision to proceed with surgery in the frail older adult requires careful deliberation to determine whether surgical management is preferable to alternative approaches (8). Older patients considering surgery should undergo a Comprehensive Geriatric Assessment (CGA)-based preoperative assessment, including an evaluation of comorbidities and functional, cognitive, and nutritional conditions (8). A number of tools are available to facilitate individualized preoperative risk assessment such as the American Society of Anesthesiologists (ASA) grade (10) or more comprehensive systems including the Physiological and Operative Severity Score for the enUmeration of Mortality and morbidity (POSSUM) (11) and its modifications (the Portsmouth or P-POSSUM), the Simplified Acute Physiology Score II (SAPS II) (12), and the Acute Physiology and Chronic Health Evaluation II (APACHE II) score (13). However, there are no geriatric-specific tools and no single instrument incorporates every important geriatric variable (8). A recent narrative review found that multidomain phenotype frailty was associated with increased risk for operative mortality (8). Therefore, for example, the Waterlow score, a tool used to stratify the risk of pressure sores and a marker of frailty has been recently compared with the ASA and the P-POSSUM to predict post-operative mortality and morbidity risk in older surgical patients obtaining good discrimination and accuracy (14). In the present article, we performed a scoping review of principal hospital- and community-based prognostic indices in older age, with a focus on drug treatment.

## Clinical Decisions in Older Adults: The Role of Frailty and Prognosis

The lack of RCTs in older subjects makes clinical decisions particularly difficult and not always appropriated, as they are based mostly on indirect data obtained from adult or younger subjects with characteristics really dissimilar from the majority of older subjects. Therefore, there is an urgent need to implement the scientific evidence in clinical practice permitting to drive more appropriately clinical decisions in older subjects, especially frail subjects with higher mortality risk. In fact, the concept of frailty, as a marker of reduced physiologic reserve, may have direct relevance to clinical care, and clearly identifies a population at greater risk of adverse health outcomes, including institutionalization, hospitalization, and death (15).

Recent epidemiological data suggested that frailty is common among critically ill patients and represents a prognostic determinant of survival and health resource utilization (16). Indeed, frail individuals are particularly susceptible to adverse drug reactions (ADRs) (17, 18), and frailty may also result in reduced treatment efficacy (19). For example, treating hypertension in healthy, robust patients older than 80 years resulted in reduced mortality and cardiovascular disease end points, but the benefits were not seen if the older patients were frail (19). The recognition of frail older subjects therefore may enable improved prognostication and shared decision-making and identify vulnerable subgroups with specific needs who might benefit from follow-up and personalized interventions. Indeed, in order to better assess the benefits and risks of different drug treatments and thus provide an health care service as close as possible to the real needs of the patients, avoiding over or underutilization of therapeutic interventions, recent guidelines recommend including life expectancy in clinical decision-making paths (20). The ultimate aim is the identification of subjects who will really benefit from a specific therapeutic intervention avoiding futile diagnostic testing (over-diagnosis) as well as surgical/invasive procedures, which do not add value and may cause harm (overtreatment o mistreatment). This action, in addition, will allow the avoidance of time- and cost-consuming medical interventions in older subjects not receiving any advantage or in fact likely displaying a higher risk of ADRs (20).

Clinical prediction rules (CPRs) (also called clinical decision rules, prediction models, prognostic tools, and risk scores) are tools designed to assist clinical decision-making. CPRs generally provide an estimate of the risk of disease, disease outcome, or the benefit of a diagnostic or therapeutic action. Recently, great attention has been paid to the proper identification of mortality prognostic tools that could help clinical decision-making in diagnostics and therapeutics to tailor appropriate intervention for the patient (21). While several risk prediction models have often been developed and validated in different populations and for different outcomes, the prognostic performance of the most popular and widely used risk models in terms of discrimination, calibration, generalizability, and reclassification is largely unknown (22). This is particularly important in advanced age due to the frequent presence of multiple comorbidities and functional deficits directly and/or indirectly affecting life expectancy.

## Multidimensional Assessment and Prognosis

There is a large and increasing body of evidence indicating that the prognosis of older patients is strongly related to the presence of concomitant diseases and to the degree of physical, cognitive, biological, and social impairment (23). The CGA, capable to effectively exploring these multiple domains of health, is indeed the multidimensional and multidisciplinary tool of choice to determine the prognosis of the functionally compromised and frail older subject (24). Initially, the “first generation” instruments for the CGA had specific targets, and were applied to specific older populations with the aim to identify and stratify the risk in predetermined clinical settings, such as older patients with depression, cognitive impairment, or physical disability. More recently, new multidimensional instruments have been introduced, creating global scores including several items that permitted to explore several different aspects of different pathologies and reassuming them in a single, standardized, and simple numerical score, assessing the global impairment of the subject that expressed the risk of health negative outcomes such as institutionalization, hospitalization, or death. Examples of these cumulative CGA-based indices are the Frailty Index-CGA (FI-CGA) (25) and the Multidimensional Prognostic Index (MPI) (26, 27) that could be useful in identifying high-risk older subjects. In particular, the FI-CGA was a 10-domain multidimensional instrument useful to assess mild, moderate, and severe frailty (25), while the 8-domain MPI was developed and validated to predict low, moderate, and severe risk of all-cause mortality (26, 27). These tools are mainly based on a list of risk factors that are mentioned to be of great importance to the concept of frailty (28), including the physical dimension (nutritional status, physical activity, mobility, strength, and energy), the psychological dimension (cognition and mood), and the social dimension (lack of social contacts and social support). These frailty/prognostic instruments are multidimensional in nature, and mostly based on a standardized CGA. A recent systematic review evaluated the clinimetric properties of 20 frailty instruments, i.e., the accuracy including discrimination and calibration, generalizability, feasibility in clinical practice, potential bias, and the possibility to be used as outcome measures (29). Unfortunately, the overall results of the assessment by using these frailty instruments suggested that they are mainly developed and validated as risk assessment tools, and not as possible outcome measures and none of these indices has been validated as predictive tool in clinical decision-making of older people (29).

## The Prognostic Indices in Older Age

### Hospital-based setting

A recent large systematic review identified, from a total number of over 21,000 titles examined, a small number of prognostic indices for mortality that meet the requirements of accuracy and calibration required to be used in a clinical setting involving hospitalized older patients (eight indices), living in nursing homes (two indices), and living in their own homes (six indices) (30). Among the eight indices selected in the hospital-based setting, only four tools estimated 1-year mortality on admission: (1) the CARING index based on four pre-specified predictors (31); (2) the Burden of Illness Score for Elderly Persons based on functional and laboratory data added to diagnoses from administrative data (32); (3) the Hospitalized Elder Longitudinal Project (HELP) survival model (33), a nomogram developed in medicine and intensive care units patients older than 80 years based on clinical information including the APACHE III scale, which requires arterial blood gas measurement; and (4) the MPI (26, 27). The MPI was the only one CGA-based predictive tool to be included in this list. None of the examined indices had accuracy excellent, i.e., a *c*-statistic value ≥0.9; moreover, for the CARING index, no *c*-statistic was reported for the external validation (31), and the Burden of Illness Score for Elderly Persons model was well calibrated at the extremes, but was less accurate in middle risk groups (32).

Among these indices used in hospital-based setting, the MPI, originally developed and validated in two independent cohorts of older patients hospitalized for acute illnesses or exacerbations of chronic diseases (26), has been identified as a tool well-calibrated (<10% of variation between the estimated and the observed mortality rates), with a good discrimination as well as with an accuracy that is maintained both at 1 month [C-index 0.76, 95% confidence intervals (CI) = 0.73–0.79] (27) and 1 year (C-index 0.75, 95% CI = 0.71–0.80) of follow-up (26) (Table 1). Among the totality of non-disease-specific prognostic indices described in this systematic review (30), the MPI was the only tool based on information obtained from a CGA exploring comprehensively health conditions (multiple comorbidities, medications, risk of pressure sores), functional (basal and instrumental activities of daily living) cognitive, and nutritional aspects as well as co-habitation status using standardized rating scales extensively validated and widely used in the aged population. The multidimensional approach as a key criterion in defining the clinical outcome in older subjects was also confirmed by studies showing the higher prognostic mortality value of MPI compared to the value displayed by the individual parameters used to build the MPI.

**Table 1:** **Clinical studies of development and validation of the Multidimensional Prognostic Index (MPI) and predictive values against different disease-specific prognostic indices**.

Disease	Patients number	Accuracy/risk AUC (95% CI) C-index, OR, or HR (95% CI)	Follow-up	Accuracy of other prognostic indices or disease-specific prognostic indices vs. MPI AUC or C-index (95% CI)	Follow-up
Acute diseases or exacerbations of chronic diseases (26)	Hospitalized Development cohort 838				
	Validation cohort 856	0.75 (0.70–0.80) 0.75 (0.71–0.80)	6 months 1 year		

Acute diseases or exacerbations of chronic diseases (27)	Hospitalized 4,088	0.76 (0.73–0.79)	1 month	m-MPI = 0.75 (0.72–0.78)	
		0.72 (0.70–0.74)	1 year	m-MPI = 0.71 (0.69–0.73)	

Acute diseases or exacerbations of chronic diseases (34)	Hospitalized Multicenter 2,033	0.76 (0.72–0.80)	1 month	FI-SOF = 0.685 (0.64–0.73) *p* < 0.0001	
				FI-CD = 0.738 (0.69–0.78) *p* < 0.0001	
				FI-CGA = 0.724 (0.68–0.77) *p* < 0.0001	
		0.75 (0.72–0.78)	1 year	FI-SOF = 0.69, 0.67–0.72, *p* < 0.0001	
				FI-CD = 0.73, 0.70–0.76, *p* < 0.0001	
				FI-CGA = 0.73, 0.70–0.75, *p* < 0.0001	

Acute diseases or exacerbations of chronic diseases (35)	Community Development cohort 7,876	MPI-SVaMA C-index (95% CI) 0.83 (0.82–0.84) 0.80 (0.78–0.80)	1 month 1 year	C-index Prognostic score: 0.82 [Lee et al. (49)]	4 years
				Combined comorbidity score: 0.79 [Gagne et al. (47)]	1 year
	Validation cohort 4,144	0.83 (0.82–0.85) 0.79 (0.78–0.80)	1 month 1 year	ASSIp prognostic index: 0.75 [Mazzaglia et al. (48)]	15 months
				PACE prognostic index: 0.74 [Carey et al. (50)]	2 years
				NHIS prognostic score: 0.75 [Schonberg et al. (51)]	5 years

Community-acquired pneumonia (37)	Hospitalized 134	0.83, 0.75–0.87	1 month	PSI = 0.71 (0.62–0.78) *p * = 0.019	
		0.79, 0.71–0.85	6 months	PSI = 0.69 (0.61–0.77) *p* = 0.035	
		0.80, 0.72–0.86	1 year	PSI = 0.75 (0.65–0.82) *p* = 0.185	

Transient ischemic attack (41)	Hospitalized 654	0.82 (0.75–0.89)	1 month		
		0.80 (0.74–0.86)	6 months		
		0.77 (0.72–0.82)	1 year		

Gastrointestinal bleeding (42)	Hospitalized 91	0.76 (0.58–0.94)	2 years	RRSS = 0.57 (0.40–0.74)	
				GBS = 0.61 (0.42–0.80)	

Liver cirrhosis (42)	Hospitalized 129	0.90 (0.85–0.96)	1 year	Child-Pugh score = 0.70 (0.52–0.88) *p* = 0.03	

Dementia (38)	Hospitalized 262	0.77 (0.73–0.84)	1 month		
		0.78 (0.72–0.83)	1 year		

Dementia (43)	Community 340	MPI score: 0–1			
		OR (95% CI) 6.50 (1.64–25.85) 9.53 (2.90–31.33)	1 year 2.2 years	Risk of hospitalization Risk of mortality	

Congestive heart failure (36)	Hospitalized 376	Men: 0.83 (0.75–0.90) Women: 0.80 (0.71–0.89)	1 month	NYHA: Men: 0.63 (0.57–0.69) *p * = 0.015; Women: 0.65 (0.55–0.75) *p * = 0.064	
				EFFECT: Men: 0.69 (0.58–0.79) *p * = 0.045; Women: 0.71 (0.55–0.87) *p * = 0.443	
				ADHERE: Men: 0.65 (0.52–0.78) *p * = 0.023; Women: 0.67 (0.49–0.83) *p * = 0.171	

Chronic kidney disease (39)	Hospitalized 786	0.70 (0.66–0.73)	1 year	eGFR = 0.58 (0.54–0.61) *p * < 0.001	

Chronic kidney disease (40)	Hospitalized 1198	C-Index (95% CI) 0.65 (0.62–0.68)	2 years	eGFR without MPI = C-index: 0.58 (0.55–0.61) *p * < 0.0001	
				Adding MPI to eGFR, C-index increased from 0.58 to 0.65 (*p * < 0.0001)	

Inoperable or metastatic solid cancer (44)	Hospitalized 160	0.91 (0.87–0.96)	6 months		
		0.87 (0.82–0.93)	1 year		

Acute diseases or exacerbations of chronic diseases (45)	Hospitalized 1,178	In-hospital mortality: C-Index 0.85 (0.79–0.91)			
		HR (95% CI): MPI-1 Reference			
		MPI-2 3.48 (1.02–11.88)			
		MPI-3 8.31 (2.54–27.19)			
		Length of stay: Mean (95%CI): MPI-1 11.29 (0.5) days; MPI-2 13.73 (1.3) days; MPI-3 15.30 (1.4) days			

*AUC, area under the curve; CI, confidence intervals; OR, odds ratio; HR, hazard ratio; m-MPI, MPI-Mini Nutritional Short-Form Examination; FI-SOF, Frailty Index from the Study of Osteoporotic Fractures; FI-CD, Frailty Index based on the Cumulative Deficit model; FI-CGA, Frailty Index based on a Comprehensive Geriatric Assessment; MPI-SVaMA, Multidimensional Prognostic Index-Standardized Multidimensional Assessment Schedule for Adults and Aged Persons; ASSIp, Assistenza Socio-Sanitaria in Italia project; PACE, Program of All-Inclusive Care for the Elderly; NHIS, National Health Interview Survey; PSI, Pneumonia Severity Score; RRSS, Rockall risk scoring system; GBS, Glasgow–Blatchford bleeding score; NYHA, New York Heart Association Functional Classification; EFFECT, Enhanced Feedback for Effective Cardiac Treatment; ADHERE, Acute Decompensated Heart Failure National Registry; eGFR, estimated Glomerular Filtration Rate*.

The MPI has been validated in over 12,000 older hospitalized patients suffering from major diseases leading to death in older subjects including gastrointestinal bleeding (42), liver cirrhosis (42), community-acquired pneumonia (37), dementia (38), congestive heart failure (36), chronic kidney disease (39, 40), transient ischemic attack (41), and cancer (44), showing also a greater predictive power for all-cause mortality than disease-specific indices for most of these conditions (36, 37, 42) (Table 1). In addition, a prospective multicenter study involving over 2,000 hospitalized older patients recruited in 20 Geriatric Units has shown that MPI was a significantly more accurate predictor of short- and long-term all-cause mortality than other three frailty indices commonly used in clinical practice (34), including the FI-CGA (25) (Table 1). Also, very recently, in a prospective study of 1,178 older patients admitted to 20 Geriatrics units, MPI score assessed at hospital admission was an independent predictor of in-hospital mortality and the length of hospital stay (45) (Table 1). Finally, the MPI in older hospitalized patients has been significantly associated to other outcomes including re-hospitalization rates and discharge destination (homes vs. nursing homes) (46), substantially improving the usefulness of this tool for resource planning purposes.

### Community-based setting

Several prognostic instruments that estimated mortality risk from 1 to 5 years have been described and validated in community-dwelling older populations. Most of these tool are based only on comorbidity score (47), while other instruments are self-reported questionnaires that evaluated functional status, age, and gender (48–50), and also the presence of multiple comorbidities (51). In community-dwelling cohorts, other indicators, more related to the pre-disability concept of frailty and to physical performance, have demonstrated a very powerful prediction of mortality, i.e., gait speed (52). Very recently, a version of the MPI, based on information collected through the Standardized Multidimensional Assessment Schedule for Adults and Aged Persons (SVaMA) has been developed and validated in two large and independent cohorts of community-dwelling older subjects (35). This MPI-SVaMA showed a very good prognostic accuracy to predict 1-month (survival C-index of 0.83) and 1-year mortality (survival C-index of 0.79) with an excellent calibration. Compared to other prognostic indices, the MPI-SVaMA differs in some crucial points: (1) the MPI-SVaMA was the only prognostic tool completely based on a CGA; (2) all data were collected directly by a multidisciplinary team, including doctors, a social worker, and a nurse; (3) no participant was excluded on the basis of incapacity of self-report; and (4) the clinical and functional scores used to calculate the prognostic index have been specifically developed and validated in older subjects. Indeed, although considering the intrinsic limitation of an indirect comparison among different prognostic instruments, the MPI-SVaMA demonstrated comparable (47, 49) or higher (48, 50, 51) discrimination as determined by the survival C-index values (Table 1). Very recently, findings from the Treviso Dementia (TREDEM) Study demonstrated that the MPI was effective also in assessing the risk of all-cause mortality and hospitalization in 340 outpatients evaluated in a tertiary care center for cognitive impairment (43) (Table 1).

Notably, the MPI showed to be an outcome measure sensitive to the antidepressant treatment in late-life major depressive disorder (MDD), suggesting an impact of selective serotonin reuptake inhibitors also on measures linked to multidimensional impairment and all-cause mortality, with a clear improvement of the MPI linked to the antidepressant treatment in older outpatients with late-life MDD (53). Furthermore, in a very recent pilot study, the integrated treatment of rivastigmine transdermal patch (RTP) plus cognitive stimulation in Alzheimer’s disease (AD) patients for 6 months improved significantly cognition, depressive and neuropsychiatric symptoms, functional status, and mortality risk assessed with the MPI in comparison with a group of AD patients receiving only RTP, confirming the possible role of this multidimensional CGA-based index as an outcome measures also in dementia (54). Therefore, the MPI score was sensitive to variations of the subject health over time in outpatients (53, 54) and hospital-based setting (45), strongly supporting the concept that considering multidimensional aggregate information could be the basis of interventions in older age.

## Prognosis and Risk–Benefit Evaluation of Drug Treatment in Older Age

Despite a great number of prognostic indices for mortality has been developed and validated, there is currently no evidence that their routine use may improve patient outcomes. Indeed, previous studies reported that the combined approach including life expectancies obtained from life tables with clinical and functional judgments by physicians can facilitate clinical decision-making in older persons (55, 56). With full access to prognostic information derived from accurate and validated predictive tools, physicians will be better equipped to make clinical decisions that are aligned with their patients’ needs in terms of safety and efficacy. For example, the decision to treat with anticoagulants older frail patients with atrial fibrillation (AF) is particularly difficult due to the high risk of serious side effects and low compliance of this treatment. Indeed, data on subjects with AF have shown that older patients with different risk of mortality are generally treated differently from each other (57). A recent retrospective observational study on almost 1300 community-dwelling frail patients aged 65 years and older with a previous hospitalization for AF, confirmed that higher MPI-SVaMA scores were associated with lower rates of warfarin treatment and higher mortality. However, a significant association between anticoagulant treatment and increased survival at 3 years of follow-up was found to be independent from age and multidimensional impairment. In fact, the analyses for heterogeneity suggested that the effect of warfarin treatment was not different among the three MPI-SVaMA groups (58).

Similarly, the clinical decision-making for the administration of statins to older patients with cardio- and cerebrovascular disease is under debate (4), with little evidence to support or refute benefit, particularly in frail older patients with comorbidity and high mortality risk. Recently, a retrospective observational study in patients aged 65 years and older with or without statin treatment, demonstrated that higher mortality risk, assessed by the MPI-SVaMA score, was associated with lower rates of statin prescription. Nonetheless, statin use was significantly associated with reduced 3-year mortality in all MPI-SVaMA-risk classes, suggesting that increased survival associated with statins in frail older patients with cardio- and cerebrovascular disease was independent of age and mortality risk (59).

## Future Research in the Field

Age distribution of patients should be representative in studies presented for marketing authorization, and collection of data from all possible sources might also be required to consolidate knowledge regarding higher-risk subpopulations. While evidence from RCTs is used to determine the efficacy of a treatment or intervention under ideal conditions, studies of observational designs are used to measure the effectiveness of an intervention in non-experimental, “real world” scenarios. Indeed, a very recent Cochrane review assessing the impact of study design on the effect measures estimated suggested that there was little evidence for significant effect estimate differences between observational studies and RCTs, regardless of specific observational study design, heterogeneity, or inclusion of studies of pharmacological interventions (60). Therefore, preliminary data from observational studies on anticoagulant and statin treatment suggested that it is time to develop clinical trials designed specifically for frail older adults (58, 59), until today not included in RCTs as a result of comorbidity or functional status. These trials might be tailored to include novel dosing schemes, alternative end points such as the impact of therapy on quality of life, cognitive or physical function, and multidimensional assessment tools to assess functional independence, family support, and also insurance coverage in older individuals (61). Unfortunately, at present, although non-disease-specific prognostic indices for older adults hold the promise of improving the targeting of interventions in advanced age, there is insufficient evidence to recommend the widespread use of prognostic indices in clinical practice (30). Large prospective trials that randomize clinicians to using these tools or not, demonstrating their impact on prognostic estimates, clinical decision-making, and patient outcomes have not been performed. Future studies are needed to independently test the accuracy of these prognostic tools in heterogeneous populations and their ability to improve clinical outcomes before their widespread use can be recommended. In conclusion, the risk of mortality may influence the effectiveness of a specific treatment in older patients. Clinicians need to consider the prognostic information obtained through well validated, accurate, and calibrated predictive tools to identify those patients who may benefit from drug treatments given with the aim of increasing survival.

## Conflict of Interest Statement

The authors declare that the research was conducted in the absence of any commercial or financial relationships that could be construed as a potential conflict of interest.
